# Experience of Spiritual Dryness and Acedia Symptoms in Seventh-Day Adventists

**DOI:** 10.1007/s10943-020-01092-7

**Published:** 2020-11-02

**Authors:** Arndt Büssing, Lorethy Starck, Klaus van Treeck

**Affiliations:** 1 Institute of Integrative Medicine, Faculty of Health, Witten/Herdecke University, Gerhard-Kienle Weg 4, 58313 Herdecke, Germany; 2 IUNCTUS - Competence Center for Christian Spirituality, Philosophical–Theological Academy, 48149 Münster, Germany; 3 Institute for Holistic Wellbeing and Resilience, Bremen, 28215 Germany; 4 Institute of Continuing Education of Seventh-Day-Adventists (IfW), Ostfildern, 30519 Germany

**Keywords:** Spiritual dryness, Acedia, Spirituality, Well-being, Seventh-day Adventists

## Abstract

In a cross-sectional survey among 626 Adventists, we investigated their perception of spiritual dryness, and its association with indicators of Acedia, well-being and emotional exhaustion. Women, younger persons and those without a specific duty within the church had significantly higher spiritual dryness scores. Spiritual dryness was predicted best by perceived *Excessive Spiritual Demands* (indicating spiritual exhaustion) and low perception of the sacred. Further predictors are Acedia’s *Difficulties in Prayer Life*, reduced well-being and emotional exhaustion. The underlying causes might be complex and thus to support persons experiencing these phases, a thorough differentiation of the underlying ‘spirits’ is required.

## Introduction

Religious persons may experience spiritual struggles, either related to the religious community, the doctrine or with God, and these struggles may affect their well-being (Exline and Rose 2013; Büssing et al. 2013; Exline et al. 2015; Stauner et al. 2016). Related to God, religious persons may specifically experience that God is distant, is not responding to their prayers, feel abandoned by God, etc., resulting in spiritual ‘dryness’ and ‘emptiness’ (Büssing et al. 2013, 2016, 2017). The perception that the prayers go unanswered was found to be often a matter of perceived distance from God (Büssing et al. 2020). The specific perception of ‘spiritual dryness’ was measured in Catholic priests (Büssing et al. 2017a, b), non-ordained Catholic pastoral workers (Büssing et al. 2016), religious brothers and sisters (Büssing 2019) and Catholic laypersons working as volunteers for disabled persons (Büssing et al. 2018a). In these groups, phases of spiritual dryness were perceived *often* to *regularly* in 12% to 14% and *sometimes* in 36% to 55%, a bit more often in religious brothers and sisters. Regression analyses revealed that low perception of the sacred in life and low sense of coherence, but also depressive symptoms and emotional exhaustion were the best predictors of spiritual dryness (Büssing et al. 2017a, b).

In theological literature, different reasons for experiences of spiritual dryness, desolation or darkness of the soul were discussed. Ignatius of Loyola (1491–1556) stated that spiritual desolation (in terms of perceived dryness in prayer and distance from God) is a tempting of the evil spirit, and a hint to change (Ignatius of Loyola 1914), while John of the Cross (1542–1591) described the ‘Dark Night of the Soul’ as a process of spiritual purification (John of the Cross, 1959, 1962). One of the ancient desert fathers, the ascetic monk Evagrius Pontikus (345–399), observed an “inertia of the heart” termed ‘Acedia’ as a cause of spiritual dryness, with symptoms of spiritual/emotional fatigue, tired or even bored negligence, and thus reduced attention in prayer (Pontikos 2007). In the life records of several saints of the Catholic church, indicators of phases of spiritual dryness were observed, i.e., Teresa of Ávila (1515–1582), John of the Cross (1542–1591), Therese of Lisieux (1873–1897) or Mother Teresa (1910–1997) (Büssing and Dienberg 2019). However, these experiences are not exclusive to Catholics, as they were observed also in persons from other religious denominations. One of the founding members of the Seventh-day Adventist (SDA) Church, Ellen G. White (1827–1915), described difficulties in her prayer life, to get in resonance with God and persons around, and symptoms of spiritual/emotional exhaustion: “None knew my labor or burden of mind as I united with the family in morning and evening devotion, and sought to lay my burden upon the great Burden Bearer. But my petitions came from a heart wrung with anguish, and my prayers were broken and disconnected because of uncontrollable grief” (White 1868). Her own struggles with depression and spiritual exhaustion are often explicated in her diary entries (Vine 2014, p. 48).

Adventists are known for better health behavior (i.e., vegetarian lifestyle, observing the Sabbath day rest, social and religious activities) because of their religiosity, and thus lower mortality (i.e., cardiovascular mortality) (Orlich et al. 2013; Morton et al. 2017). The pathways were investigated by Morton et al. (2017) who found that US American SDAs’ mortality was predicted by religious engagement, but mediated by religious support, emotionality and lifestyle, while their church activity had both a direct effect and an indirect effect through religious support, emotionality and lifestyle. So far there was no clear evidence that being a SDA would reduce the risk to experience phases of depression or spiritual struggles, although it was shown that religious involvement in general is related to better mental health outcomes—however, with differences between women and men (Maselko and Kubzansky 2006) and ethnicity (Taylor et al. 2004).

For SDAs, daily prayer and an unbroken relationship with God are the core of their spirituality (SDA Fundamental Belief Nr. 11; White 1892). Further, church attendance and Sabbath keeping are regarded as an important source of spiritual and social well-being. Therefore, an active spiritual life may result in growing love and confidence in God, and inner peace and rest (White 1892). However, what happens when the specific persons are not able to perceive this, when their spiritual life becomes difficult and ‘dry’ and their relation to God faint?

We therefore intended to analyze the experience of spiritual dryness in SDA, its association to indicators of Acedia (namely perceived excessive spiritual demands by God and difficulties in prayer life), whether and how these experiences are related to experiential spirituality on the one hand, and to the specific religious lifestyle of SDA (i.e., prayer life, strictness of observing the Sabbath, strictness of vegetarian lifestyle) and finally associations with indicators of low well-being and emotional exhaustion.

## Materials and Methods

### Study Participants

Members of the SDA Church Inter-European Division (Bern) were invited by emails to the European coordinators, regional groups, Facebook groups and SDA information journals to participate in an anonym online survey with standardized measures. By filling in the anonymous questionnaire, the practitioners consented to participate. Neither concrete identifying personal details nor IP addresses were recorded to guarantee anonymity.

Due to this recruitment method, we were unable to calculate a precise response rate. However, among 918 German language and Francophone language persons who visited the website, 27% did not start the survey. Among these 669 starters, 6% did not responded to the Spiritual Dryness Scale, leaving 626 responder.

### Measures

The questionnaire asks for gender, age, position within the church, how long one is member of the SDA. All further standardized measures will be described in the following.

#### Spiritual Dryness

Perceptions of ‘spiritual dryness’ were assessed with the Spiritual Dryness Scale (SDS). It uses primarily six items which have good internal consistency (Cronbach’s α = 0.87) (Büssing et al. 2013). Specific statements refer to the feelings that God is distant, that one’s prayers go unanswered, of being “spiritually empty” or not able to give any more (in terms of spiritual exhaustion) and, finally, feelings of being abandoned by God. The items of this instrument were formulated in order to fit into the daily life experiences of religious individuals. For this analysis, we added a further item which asks for a “deep longing for God” (item SDS0). Response options on a Likert scale were *not at all* (1), *rarely* (2), *occasionally* (3), *fairly often* (4) and *regularly* (5). The SDS scores are mean scores and represent the perceived lack/shortage.

#### Acedia Symptoms

To measure symptoms related to Acedia in a wider context, we referred to items which were previously used in a study among religious brothers and sisters (Büssing 2019). Ten items were used, among them two were intended as informative items (“My prayer life is rich and fulfilling,” “In prayer I am focused and present before God”), while the other eight items address experiences of difficulties in prayer life (in terms of inattentiveness and distance) and excessive spiritual demands (in terms of perceived overcharging demands referred to God). Examples of the former topic are “I am more passive in prayer and without any inner involvement,” “My prayer life doesn’t excite me so much anymore,” “I really enjoy only a little in my spiritual life,” “I don’t really care whether I find God in prayer or not,” while the latter topic was addressed by items such as “What God asks of me is more than I can give,” “What God asked of me is just too much,” “I really don’t know what God wants from me,” “Somehow, everything got too much for me.” Reliability analysis of these items will be presented in this article. Response options on a Likert scale were *not at all* (1), *rarely* (2), *occasionally* (3), *fairly often* (4) and *regularly* (5).

#### Spiritual Practices and Behaviors

Frequency of spontaneous prayer (‘privately’; apart from public worship/church service, etc.) was measured with a five-grade scaling (*several times per day*; *daily*; *on several days*; *on some days*; *rarely/nearly never*), while the frequency of church attendance was measured with a four-grade scale (*at least once per week*; *several times per month*; *once per month*; *less than once per month*). Participants were also asked how strict they observe the Sabbath (apart from public worship) and how strict they follow a vegetarian/vegan lifestyle, using a four-grade scaling (*very strictly*; *quite strictly*; *not that strict*; *not at all strictly*).

#### Transformative Spirituality

To address transformative aspects of spirituality in terms of attitudes and subsequent behaviors, we used the Franciscan-inspired Spirituality (FraSpir) questionnaire. This instrument uses 26 items and differentiates four subscales: *Live from the Faith/Search for God*; *Peaceful attitude/Respectful treatment; Commitment to Disadvantaged and Creation; Attitude of Poverty* (Büssing et al. 2017b). The internal reliability of these factors ranges from Cronbach’s alpha 0.79–0.97. All items were scored on a five-point scale from disagreement to agreement [0—*does not apply at all*; 1—*does not truly apply*; 2—*half and half* (*neither yes nor no*); 3—*applies quite a bit*; 4—*applies very much*].

#### Perception of the Sacred

The Daily Spiritual Experience Scale (DSES) was developed as a measure of a person’s perception of the sacred in daily life, and thus the items measure experience rather than particular beliefs or behaviors (Underwood and Teresi 2002; Underwood 2011). Here, we used the six-item version (DSES-6; Cronbach’s alpha = .91) which uses specific items such as feeling God’s presence, God’s love, desire to be closer to God (union), finding strength/comfort in God, being touched by beauty of creation (Underwood and Teresi 2002). The response categories from 1 to 6 are *many times a day*, *every day*, *most days*, *some days*, *once in a while* and *never/almost never*. Item scores were finally summed up.

#### Awe and Gratitude

Awe/gratitude as an indicator of experiential spirituality was measured with the seven-item gratitude/awe questionnaire (GrAw-7) (Büssing et al. 2018b) which has good psychometric properties (Cronbach’s alpha = 0.82). This measure has a clear focus on the experiential aspects of being moved and touched by certain moments and places/nature, related reactions of pausing with daily activities and on the subsequent feelings of awe and gratitude. Specific items are, “I stop and then think of so many things for which I’m really grateful,” “I stop and am captivated by the beauty of nature,” “I pause and stay spellbound at the moment” and “In certain places, I become very quiet and devout.” It is a measure of all items scored on a four-point scale (0—*never*; 1—*seldom*; 2—*often*; 3—*regularly*), referred to a 100% scale. The resulting mean values thus range from 0 to 100.

#### Well-being

Well-being was assessed with the five-item WHO-Five Well-Being Index (WHO-5) (Bech et al. 2013). Representative items are “I have felt cheerful and in good spirits” or “My daily life has been filled with things that interest me.” The intensity of feelings refers to the last 2 weeks and was scored with a six-step grading scale ranging from *at no time* (0) to *all the time* (5). Here, the sum scores ranging from 0 to 25 were reported. Scores < 13 may indicate depressive states.

#### Under Pressure and Emotional Exhaustion

Perceived emotional exhaustion and feelings of being ‘under pressure’ (either by stress, anxiety, etc.) were measured with two visual analog scales (VAS) ranging from *not at all* (0) to *extremely* (100).

### Statistical Analyses

Descriptive statistics, analyses of variance (ANOVA), first-order correlations (Spearman rho) and regression analyses as well as internal consistency (Cronbach’s coefficient α) and factor analyses (principal component analysis using Varimax rotation with Kaiser’s normalization) were computed with SPSS 23.0. Cluster analysis was performed with SPSS 25.0.

Given the exploratory character of this study, the significance level of ANOVA and correlation analyses were set at *p* < 0.05. With respect to classifying the strength of the observed correlations, we regarded *r* > .5 as a strong correlation, an *r* between .3 and .5 as a moderate correlation, an *r* between .2 and .3 as a weak correlation and *r* < .2 as negligible or no correlation.

## Results

### Description of the Sample

We have analyzed data from 626 SDA from Germany (54%), Austria (27%) and the Francophone countries France, Belgium and Luxembourg (19%). Among them, 45% were women and 55% men with a mean age of 50.1 ± 15.3 years. Most had grown up as SDA (69%), with a mean number of 29.1 ± 16.1 years as a SDA. Within the sample, 13% were pastors, 12% elder, 8% deacons, 45% had other duties within the church and 21% had none (Table 1).

**Table 1:** Description of the sample (*N* = 626)

Gender (%)	
Women	45
Men	55
Age (years)	50.1 ± 15.3 [14–99]
Country (%)	
Germany	54
Austria	27
France, Belgium and Luxembourg	19
SDA since … (years)	29.1 ± 16.1 [0–74]
Since … years either fill-time of voluntarily engaged in the free-church (years)	22.9 ± 15.1 [0–74]
Raised as SDA (%)	
Yes	69
No	31
Duty within the free-church (%)	
Pastor	13
Elder	12
Deacon	8
Other duties	45
None duty	21
How often do you pray privately? (%)	
Several times per day	58
Daily	27
At several days	8
At some days	5
Seldom/rather never	2
How often going to church? (%)	
At least once per week	72
Several times per month	19
Once per month	2
Less than once per month	7
How strict do you hold the Sabbath? (%)	
Very strict	24
Somewhat strict	49
Not that strict	21
Not at all strict	6
How strict do you follow a vegetarian lifestyle? (%)	
Very strict	16
Somewhat strict	23
Not that strict	23
Not at all strict	38
Live from the faith/search for God (FrSpir)	3.2 ± 0.6 [0–4]
Peaceful attitude/respectful treatment (FrSpir)	3.0 ± 0.6 [0–4]
Commitment to disadvantaged and creation (FraSpir)	2.6 ± 0.9 [0–4]
Attitude of poverty (FraSpir)	2.6 ± 0.8 [0–4]
Perception of the sacred (DSES-6)	4.3 ± 1.0 [1–6]
Gratitude/awe (GrAw-7)	66.7 ± 18.0 [0–100]
Under pressure (VAS)	45.7 ± 28.6 [0–100]
Emotional exhaustion (VAS)	37.9 ± 30.6 [0–100]
Well-being (WHO-5)	14.8 ± 5.4 [0–25]

Most were praying privately at a daily level (85%), and 72% were attending church each week. Strictness to hold the Sabbath was variable, 24% are very strict and 49% somewhat strict (Table 1). Most are not that strict with a vegetarian/vegan lifestyle (61%). The study participants regarded *Live from the Faith/Search for God* and also *Peaceful attitude/Respectful treatment* (of others) as relevant and scored this high on both subscales. Also their *Commitment to Disadvantaged and Creation* and an *Attitude of Poverty* scored in the high-range of relevance (Table 1). With respect to the more experiential indicators of spirituality, perception of the sacred in their life (DSES-6) and also awe and subsequent gratitude (GrAw-7) scored in the mid-range.

Feelings to be ‘under pressure’ scored moderately high (mean score: 45.7 ± 28.6) with large variance. Emotional exhaustion was perceived by several of them at a ‘somewhat’ level (mean score: 37.9 ± 30.6). Their well-being (WHO-5) scored in the lower range (14.8 ± 5.4) (Table 1).

### Experience of Spiritual Dryness

Within the sample, 16% experienced phases of spiritual dryness *often* to r*egularly*, 38% *occasionally*, 34% *rarely* and 11% *not at all* (Table 2). 12% experienced *often* to r*egularly* that their prayers go unanswered, and 11% had the feeling that God is distant from them regardless of their efforts to draw close to him. This does not necessarily mean they feel that God has abandoned them completely; only 4% perceived this *often* to r*egularly*, 23% at least occasionally. A ‘spiritual emptiness’ was perceived by 16% *often* to *regularly*, while 21% do know the associated feeling of not being able to (emotionally) give any more. A deep longing for God was nevertheless perceived by most of them *often* to *regularly* (74%), 19% *occasionally* and 8% only *rarely* or *not at all* (Table 1).

**Table 2:** Response rates

	Not at all (%)	Rarely (%)	Occasionally (%)	Fairly often (%)	Regularly (%)	Score (1–5)
Item phrasings: spiritual dryness						
I have the feeling that God is distant from me, regardless of my efforts to draw close to him	34	33	23	8	3	2.13 ± 1.05
I have the feeling that God has abandoned me completely	66	22	9	3	1	1.52 ± 0.85
I experience times of ‘spiritual dryness’	11	34	38	12	4	2.65 ± 0.99
I have the feeling that I am ‘spiritually empty’	26	32	29	10	4	2.34 ± 1.08
I have the feeling that my prayers go unanswered	28	34	26	8	4	2.27 ± 1.08
I know the feeling of not being able to give any more	16	31	31	15	6	2.64 ± 1.11
*I feel a deep longing for God	3	5	19	39	35	3.97 ± 1.00
Item phrasings: Acedia						
*Ac1 My prayer life is rich and fulfilling	7	14	34	35	11	3.31 ± 1.06
*Ac4 In prayer I am focused and present before God	1	8	22	47	22	3.79 ± 0.92
Ac2 I am more passive in prayer and without any inner involvement	23	41	24	10	1	2.25 ± 0.97
Ac5 My prayer life doesn’t excite me so much anymore	26	27	15	18	4	2.45 ± 1.16
Ac6 I don’t really care whether I find God in prayer or not	77	12	7	3	1	1.40 ± 0.85
Ac9 I really enjoy only a little in my spiritual life	36	31	20	11	3	2.14 ± 1.11
Ac12 What God asks of me is more than I can give	50	24	17	6	4	1.89 ± 1.11
Ac13 What God asked of me is just too much	52	24	17	5	2	1.80 ± 1.01
Ac14 I really don’t know what God wants from me	30	26	28	12	5	2.37 ± 1.17
Ac15 Somehow, everything got too much for me	40	27	18	9	6	2.13 ± 1.20

*Informative items not used in the respective scales

### Acedia Symptoms

With respect to their prayer life, most would see it as rich and fulfilling (46% *often* to *regularly*; 21% *rarely* or *not at all*) and most regard themselves as focused and present during prayer (69% *often* to *regularly*; 9% *rarely* or *not at all*) (Table 2). In contrast, 22% stated that their prayer life does not ‘excite’ them so much anymore (*often* to *regularly*), and 11% were rather passive during prayer and without inner involvement (*often* to *regularly*). Similarly, 14% stated that they *often* to *regularly* can really enjoy only a little in their spiritual life. An excessive spiritual demand was experienced by up to 10% *often* or even *regularly* (items Ac12 and Ac13). 17% stated that they *often* to *regularly* do not know what God wants from them, indicating emotional/spiritual distance. Finally, 15% reported that “somehow, everything got too much” for them, indicating spiritual exhaustion. Nevertheless, while several do have these perceptions, only 4% would state that they *often* to *regularly* do not care whether they find God in prayer or not (Table 2).

In order to further analyze the impact of these perceptions and behaviors related to Acedia symptoms, we performed a reliability and factor analysis with these Acedia items to combine them as a common factor. Among these ten items, two were intended as informative items (Ac1, Ac4), while the eight other address experiences of difficult prayer life and excessive spiritual demands. With a Kaiser–Meyer–Olkin value of 0.83 and a significant Bartlett test (*p* < 0.0001), these eight items are suitable for factor analysis. Explorative factor analysis (principal component analysis with Varimax rotation and Kaiser normalization) revealed two main factors which would explain 62% of variance (Table 3). The four-item factor *Excessive Spiritual Demands* has good (Cronbach’s alpha = .81) and the four-item factor *Difficulties in Prayer Life* has acceptable internal consistence (Cronbach’s alpha = .75). With Cronbach’s alpha = .84, the eight-item Acedia scale has good internal consistence, too.

**Table 3:** Factor and reliability analysis of the Acedia module

	Corrected item—scale correlation	Cronbach’s alpha when item is deleted (alpha = .841)	Factor loading	
			1	2
*Factor 1: excessive spiritual demands* Eigenvalue = 3.8; Cronbach’s alpha = .809				
Ac13 What God asked of me is just too much	.678	.809	.884	
Ac12 What God asks of me is more than I can give	.567	.823	.829	
Ac15 Somehow, everything got too much for me	.606	.818	.738	
Ac14 I really don’t know what God wants from me	.571	.822	.612	.342
*Factor 2: Acedia—difficulties in prayer life* Eigenvalue = 1.2; Cronbach’s alpha = .753				
Ac5 My prayer life doesn’t excite me so much anymore	.597	.819		.779
Ac6 I don’t really care whether I find God in prayer or not	.370	.843		.725
Ac2 I am more passive in prayer and without any inner involvement	.526	.828		.719
Ac9 I really enjoy only a little in my spiritual life	.660	.810	.507	.587

Extraction: Principal component analysis (Varimax rotation with Kaiser normalization); rotation converged in three iterations

### Expression of Spiritual Dryness and Acedia Symptoms in the Sample

Women had significantly higher spiritual dryness and Acedia scores (particularly *Excessive Spiritual Demands*, but not *Difficulties in Prayer Life*) than men, while their perception of the sacred was similar (Table 4). With respect to the age cohorts, spiritual dryness and Acedia symptoms were significantly higher in younger SDAs compared to the older persons, while their perception of the sacred was significantly lower.

**Table 4:** Expression of SDS, Acedia and DSES

	Spiritual dryness (SDS-6)	Acedia score	Acedia: excessive spiritual demands	Acedia: difficulties in prayer life	Perception of the sacred (DSES-6)
All					
M	2.26	2.06	2.06	2.06	25.61
SD	0.82	0.74	0.91	0.78	6.20
*Gender*					
Women					
M	2.37	2.15	2.26	2.06	25.44
SD	0.79	0.78	0.96	0.78	6.25
Men					
M	2.17	1.98	1.91	2.06	25.75
SD	0.82	0.70	0.84	0.78	6.17
F value	8.68	8.13	23.19	0.00	0.37
*p* value	0.003	0.005	< 0.0001	n.s.	n.s.
*Age cohorts*					
< 41 years					
SD	2.46	2.23	2.30	2.18	24.53
M	0.84	0.79	0.99	0.79	5.89
41–60 years					
SD	2.24	2.02	2.01	2.04	25.64
SD	0.76	0.70	0.84	0.74	6.31
> 60 years					
M	2.05	1.93	1.91	1.96	26.85
SD	0.83	0.75	0.88	0.83	6.05
*F* value	11.15	7.58	8.84	3.28	5.86
*p* value	< 0.0001	0.001	< 0.0001	0.038	0.003
*Duty in the church*					
Pastor					
M	2.11	1.90	1.78	2.03	27.26
SD	0.68	0.58	0.72	0.71	5.21
Elder					
M	1.99	1.93	1.91	1.94	27.51
SD	0.72	0.66	0.77	0.73	5.34
Deacon					
M	2.13	1.85	1.89	1.81	28.11
SD	0.70	0.65	0.82	0.61	5.07
Other duties					
M	2.27	2.07	2.08	2.06	25.27
SD	0.79	0.75	0.92	0.76	5.97
No duty					
M	2.56	2.31	2.37	2.26	23.35
SD	0.94	0.82	1.01	0.90	7.01
*F* value	7.94	6.42	6.95	4.08	11.05
*p* value	< 0.0001	< 0.0001	< 0.0001	0.001	< 0.0001

Interestingly, spiritual dryness was highest in persons without any duty within the SDA church and lowest in elders, pastors and deacons, while the sacred in life was inversely perceived (Table 4). Similarly, Acedia symptoms were highest in persons without any duties and lowest in deacons and pastors.

### Longing for God’s Closeness

As stated above, even though they may experience these phases of spiritual dryness, most have a longing for God. This longing can be categorized with respect to its frequency as *regularly* (34% high), *often* (39% moderately) and *occasionally/seldom/not at all* (27% low). As shown in Table 5, there were no significant differences between these longing categories with respect to the experience of spiritual dryness, while Acedia’s *Difficulties in Prayer Life* particularly was significantly higher in persons with a low longing for God. The same is true for their perception of the sacred, which was significantly lower in persons with low longing compared to those with moderate and high longing for God’s closeness. Acedia’s *Excessive Spiritual Demands* was not significantly differently perceived in these three groups.

**Table 5:** Longing for God and its association with spiritual dryness and Acedia symptoms

Deep longing for God (SDS0)	Spiritual dryness (SDS-6)	Acedia score	Acedia: excessive spiritual demands	Acedia: difficulties in prayer life	Perception of the sacred (DSES-6)
All perceptions (*n* = 619)					
M	2.26	2.06	2.06	2.06	4.27
SD	0.81	0.74	0.90	0.78	1.03
Not at all/seldom/occasionally (*n* = 165)					
M	2.29	2.19	2.13	2.26	3.89
SD	0.92	0.77	0.94	0.82	1.14
Often (*n* = 240)					
M	2.33	2.06	2.08	2.05	4.24
SD	0.73	0.71	0.88	0.73	0.94
Regularly (*n* = 214)					
M	2.17	1.95	1.99	1.92	4.60
SD	0.81	0.72	0.89	0.76	0.94
*F* value	2.36	4.84	1.20	9.96	23.80
*p* value	n.s.	.008	n.s.	< .0001	< .0001

### Correlation Analyses

To analyze how the experience of spiritual dryness is related to indicators of Acedia, and how both are related to indicators of spirituality on the one hand and indicators of reduced well-being on the other hand, we performed correlation analyses (Table 6).

**Table 6:** Correlation analyses

	Spiritual dryness (SDS-6)	Acedia score	Acedia: excessive spiritual demands	Acedia: difficulties in prayer life
Spiritual dryness (DSES-6)	1.000			
Acedia score	.723**	1.000		
Acedia: excessive spiritual demands	.658**	.880**	1.000	
Acedia: difficulties in prayer life	.618**	.855**	.527**	1.000
*Indicators of spirituality*				
Perception of the sacred (DSES-6)	− .641**	− .606**	− .479**	− .589**
Gratitude/awe (GrAw-7)	− .395**	− .387**	− .290**	− .402**
Deep longing for God (SDS0)	− .036	− .110**	− .048	− .156**
Live from the Faith/search for God	− .428**	− .475**	− .352**	− .489**
Peaceful attitude/respectful treatment	− .208**	− .244**	− .225**	− .214**
Commitment to disadvantaged and creation	− .270**	− .278**	− .284**	− .206**
Attitude of poverty	− .229**	− .221**	− .186**	− .210**
Frequency private prayer	.339**	.398**	.260**	.436**
Frequency Church attendance	.215**	.248**	.229**	.212**
Strictness to hold the Sabbath	.276**	.289**	.221**	.285**
Strictness of vegetarian lifestyle	.153**	.185**	.135**	.198**
*Indicators of well-being*				
Under pressure (VAS)	.371**	.345**	.354**	.245**
Emotional exhaustion (VAS)	.500**	.487**	.489**	.356**
Well-being (WHO-5)	− .543**	− .478**	− .482**	− .359**

**p < 0.001 (Spearman rho); moderate to strong correlations are highlighted (bold)

Spiritual dryness was strongly interrelated with both Acedia subscales. Therefore, the correlations with the other measures are rather similar. Both are strongly inversely related to perception of the sacred and moderately negatively with *Live from the Faith/Search for God*, gratitude/awe and frequency of prayer life. Both were moderately to strongly related to emotional exhaustion, feelings of being ‘under pressure’ and reduced well-being.

Specific aspects of SDA’s spirituality such as frequency of church attendance and strictness to hold the Sabbath were weakly related to spiritual dryness and both Acedia subscales, while the strictness of a vegetarian lifestyle was marginally only related (Table 6). Further, *Peaceful attitude/Respectful treatment*, *Commitment to Disadvantaged and Creation* and an *Attitude of Poverty* were weakly negatively associated, too.

In contrast, their longing for God was not relevantly related to spiritual dryness, and marginally negatively only with lower Acedia scores (Table 6).

### Predictors of Spiritual Dryness

So far there were several significant associations between spiritual dryness on the one hand and indicators of spirituality and reduced well-being on the other hand; we further observed differences related to socio-demographic data. To analyze which of these variables would predict spiritual dryness, we performed regression analyses in different steps. First, we analyzed the influence of gender, age cohorts, recruiting country (Germany versus other), or being without a duty within the church. However, these four variables would predict only 9% of variance and are thus less relevant; here ‘no duty’ (Beta = 0.15, *T* = 3.64, *p* < 0.0001) and country (Beta = 0.13, *T* = 3.21, *p* = 0.001) were the best, but weak predictors. In the following regression models, none of these were of significant relevance, and they were thus excluded.

As shown in Table 7, the next model tested involved feelings of being ‘under pressure’ (VAS), emotional exhaustion (VAS), well-being (WHO-5), *Live from the Faith/Search for God* (FraSpir), perception of the sacred (DSES-6) and gratitude/awe (GrAw-7). This model would explain 56% of variance. Here, perception of the sacred was the best predictor, followed by emotional exhaustion and low well-being; gratitude/awe had a marginal effect, while feeling ‘under pressure’ and *Live from the Faith/Search for God* had no significant effect.

**Table 7:** Predictors of spiritual dryness in SDA

	Model 1: *R*^2^ = .56			Model 2: *R*^2^ = .68		
	*B*	*T*	*p*	*B*	*T*	*P*
Constant		27.289	< .0001		12.247	< .0001
Under pressure (VAS)	.032	.815	.415	.016	.461	.645
Emotional exhaustion (VAS)	.208	4.676	< .0001	.098	2.500	.013
Well-being (WHO-5)	− .128	− 3.286	.001	− .087	− 2.587	.010
Live from the Faith/search for God (FraSpir)	− .052	− 1.487	.137	.004	.118	.906
Perception of the sacred (DSES-6)	− .568	− 13.367	< .0001	− .358	− 8.920	< .0001
Gratitude/awe (GrAw-7)	.078	2.097	.036	.044	1.378	.169
Acedia: excessive spiritual demands	–	–	–	.329	10.377	<.0001
Acedia: difficulties in prayer life	–	–	–	.187	5.775	< .0001

Then, both Acedia subscales were added to the regression model, which now would explain 68% of variance. Here, the best predictors were Acedia’s *Excessive Spiritual Demands* and low perception of the sacred and further Acedia’s *Difficulties in Prayer Life*. Additional weak predictors were emotional exhaustion and low well-being. Acedia’s *Excessive Spiritual Demands* alone would predict 49% of variance, as verified with a stepwise regression analysis; perception of the sacred would add 14% of explained variance and Acedia’s *Difficulties in Prayer* further 2%, the other two variables would finally add further 2% of explained variance.

### Cluster Analyses

On basis of the categorical variable spiritual dryness and the continual variables well-being (WHO-5), perception of the sacred (DSES-6), Acedia’s *Excessive Spiritual Demands*, Acedia’ *Difficulties in Prayer Life* and emotional exhaustion (VAS), we performed a *Z*-valued two-step cluster analysis (*N* = 602). The predictor influences for spiritual dryness (1.00) are Acedia’s *Excessive Spiritual Demands* (0.32), perception of the sacred (0.28), Acedia’s *Difficulties Prayer Life* (0.27), well-being (0.17) and emotional exhaustion (0.14).

We found three clusters of SDAs’ spiritual dryness experience (Table 8). The sharpest differences were between Cluster 1 and Cluster 3, with high scores for spiritual dryness and both Acedia subscales, low well-being, low perception of the sacred and high emotional exhaustion in Cluster 1 (31%), while Cluster 3 (38%) shows high perception of the sacred, high well-being, low emotional exhaustion, low spiritual dryness and low Acedia. The respective scores of these variables were in an intermediate range within Cluster 2 (31%).

**Table 8:** Three clusters of spiritual dryness experience among SDA

	Perception of the sacred (DSES-6)		Acedia: excessive spiritual demands		Acedia difficulties prayer life		Well-being (WHO-5)		Emotional exhaustion (VAS)		Spiritual dryness (SDS)	
	Mean	SD	Mean	SD	Mean	SD	Mean	SD	Mean	SD	Mean	SD
Cluster 1	20.55	5.82	2.87	0.93	2.69	0.76	11.32	5.41	56.76	26.92	3.23	0.27
Cluster 2	26.16	4.48	1.92	0.66	1.96	0.55	14.52	4.82	37.19	29.02	2.23	0.19
Cluster 3	29.56	4.35	1.50	0.50	1.58	0.54	17.80	3.96	23.06	26.21	1.48	0.59
Mean	25.70	6.16	2.06	0.91	2.05	0.77	14.77	5.42	37.92	30.65	2.26	0.82

## Discussion

The participants in the study describe themselves as Adventists who go to church often, pray privately several times per day and hold the Sabbath at least somewhat strict; they live from their faith and they have a deep longing for God. However, their average well-being scores are in a lower range, and they moderately feel under pressure. In line with this, several experience phases of spiritual dryness—to a similar extent as the Catholic participants of the pastoral ministry study in German dioceses (Frick et al. 2016). Significantly more women than men, younger persons than older ones and persons without a specific duty within the SDA church had higher spiritual dryness scores. Also, the indicators of Acedia, perceived *Excessive Spiritual Demands* and *Difficulties in Prayer Life*, were reported by several of the investigated persons; again, significantly more often by women, younger persons and persons without a duty within the church. Both experiences were in fact strongly interconnected. Predictor analyses revealed that spiritual dryness was predicted best by Acedia’s *Excessive Spiritual Demands* (which indicates a spiritual exhaustion related to the perception that God asks more than one is able to give, or an uncertainty what God “wants from me”) and low perception of the sacred in life. Further predictors are Acedia’s *Difficulties in Prayer Life* (which is perceived as less “exciting,” associated with reduced joy in spiritual life, and thus passivity during prayer), and to a lower extent also reduced well-being and emotional exhaustion. One might find three clusters of spiritual dryness experience which are characterized by the following polarities: high perception of the sacred, high well-being and low emotional exhaustion, and low spiritual dryness and Acedia scores on the one hand (38%), and low perception of the sacred, high emotional exhaustion and very low well-being (indicating depressive states), and high spiritual dryness and Acedia scores on the other hand (31%). Cluster 2 is characterized by quite high perception of the sacred, moderate emotional exhaustion and rather low well-being, low Acedia scores and moderate spiritual dryness scores (31%).

These results are remarkable in the light of the research of the Adventist church historian George Knight. Knight (1989) describes three forms of Adventist spirituality: (1) “Sadventists” who have not yet experienced the joy of salvation and spiritual life; (2) “Madventists” who struggle for transformation and character perfection, and who are focused on their own actions in faith and uphold the law and (3) “Gladventists” who are filled with the joy, love and kindness of Jesus and are changed by it. According to Knight, Adventists are still caught up (negatively) in this conflict between the ‘right teaching’ (knowledge) and ‘compliance with the law’ (works) as well as ‘freedom of belief’ (grace) as a refreshing experience (see Acts, Romans and Galatians; see also Johnsson 2017, who shares this concern in a review of the last General Conference of SDA in 2015). Ellen White, an Adventist founder, said in 1888 about this polarization between law and grace, “*Let the law take care of itself. We have been at work on the law until we get as dry as the hills of Gilboa…. Let us trust in the merits of Jesus*.” “*The love of Christ in the heart*,” she concluded, “*will do more to convert sinners than all the sermons you can preach. What we need is to get the love of Christ*.” She uses the metaphor “as dry as the hills of Gilboa” (referring to Samuel 1:22) quite often in her articles and books to describe the spiritual dryness of church members, pastors and the Church as a whole. Her literary works from the end of the 19th century (*Steps to Christ*, *The Desire of Ages*, *Thoughts from the Mount of Blessings*, *The Ministry of Healing*) are fully dedicated to overcoming the spiritual dryness in Adventism through a balanced understanding of law versus grace. It shows how Christians, through the dedication of their lives to Jesus, can come to a vigorous development of the physical, mental, social and spiritual powers of man. However, these necessary processes might have slowed down in some of today’s SDA, calling for an individual and organizational rethinking.

We are not sure whether the three clusters described above could be related to Knight’s three ‘types’ of SDAs’ spirituality. However, neither the cluster data nor the SDS itself is intended as a diagnostic tool (lacking clear cutoffs), but to start awareness of an often neglected topic. Religious persons may experience difficult phases during their spiritual life, even as they long to be closer to God. These could be a matter of too high expectations, overestimation of own spiritual maturity, or lacking confidence in God’s work (even within dark times in life) on the one hand, but also difficulties with others in the community, lacking recognition by ‘superiors,’ lacking abilities or opportunities to engage for the church on the other hand—but also low coherence in life and states of depression. All these reasons point to the fact that there is a quite large group of SDAs analyzed in this study who require spiritual support. However, in most cases, persons concerned have no partner to talk about their spiritual struggles, as they are afraid to be identified as ‘poor in faith.’ Here is an important chance for pastors, elders and deacons to provide low-threshold offers which encourage discussions about these difficulties in spiritual life. These difficulties are not necessarily an individual ‘failure,’ as they are regarded as an inevitable necessity by the theologian Karl Rahner (1993). The problems may arise when one intends to define God in terms of ‘how’ and ‘what’ God is, and these ‘false’ thoughts do not meet God’s complete ‘otherness,’ which may be perceived as distance from God. Only when one is able to fully open oneself to this incomprehensibility of God, one may perceive a vanishing of the world (which is experienced as “darkness”) as the onset of the rise of God in the soul (“light”) (with reference to Rahner 1993). One may rather consider whether those who are too sure about their good relation with ‘their’ (private) God and who have never experienced phases of doubt, struggles or dryness are the more problematic ones. It might be, as suggested by Knight (1989) that some “have been so busy preaching the law and cleansing their soul temple of every defilement so that they can somehow make it through the time of trouble that they have failed to find Christ and Christianity.” However, this is surely not specific of SDAs, but of several Christian denominations.

## Limitations

We do not assume that our sample is representative of all SDAs in Europe. Here, we have focused on SDAs from Germany (54%), Austria (27%) and the Francophone countries France, Belgium and Luxembourg (19%), which are underrepresented in this sample. Cultural peculiarities and characteristics might play a further role in how one perceives spiritual struggles. These groups differ significantly also with respect to the duration of being a baptized SDA (*F* = 8.2, *p* < 0.001), but also with respect to their spiritual dryness scores (*F* = 8.6, *p* < 0.0001) and their well-being (*F* = 9.0, *p* < 0.0001). Participants from Germany had significantly lower well-being and higher spiritual dryness. However, it was not the aim of this study to compare persons from different countries, but to generally analyze this neglected topic. Nevertheless, participants from other countries are needed and currently invited. Moreover, only SDAs with internet access were able to participate in this survey. Further, we are not sure about the ‘passive’ members which are difficult to access and invite. Most participants of this study have a specific duty within the church, only 21% do not; this might indicate a bias in favor of the active members.

With these data from a cross-sectional survey, we cannot analyze spiritual development processes. Some have already experienced these phases of spiritual dryness or currently experiencing these, others not (yet). Within the sample of 626 persons, 92% responded to an additional item which should be responded only when phases of spiritual dryness were experienced at least sometimes or more frequently. Among these 578 persons, 15% stated that they *did not* find ways to cope with spiritual dryness, 30% *sometimes*, 37% *often* and 19% *regularly* (indicating that most do have found ways to cope). Moreover, 11% did not experience spiritual dryness at all. The processual activity thus cannot be taken into account for data interpretation.

In the early centuries of Christianity, Acedia was regarded as one of the ‘deadly sins’ in terms of spiritual apathy (Jamison 2008), as bored weariness and not caring for the divine good, and thus personal failure. For this analysis, Acedia was operationalized as perceptions and behaviors all persons may have in their spiritual life: The negative attributions of failure and guilt were not implied, while attitudes of negligence and weakness seem to be are more acceptable. Thus, this specific operationalization should not be seen as a limitation, but as an attempt to minimize false negative responses due to shame and religious desirability.

## Conclusion

The topics of spiritual dryness and related indicators of Acedia were for the first time systematically analyzed also in SDAs. Despite their strong reference to performing a deeply religious life with regular church attendance, private praying, observing the Sabbath and living from their faith, several of them nevertheless experience phases of spiritual dryness just like other religious persons, too. Some measure of insecurity of what God “wants” from them, and a loss of “excitement” in their prayer life was found in relevant proportion. Perceived *Excessive Spiritual Demands* and low perception of the sacred in their life were the best indicators of the frequency of spiritual dryness. For some, this might indicate an inability to perceive God despite a deep longing, while for others, it seems to be a matter of emotional exhaustion and reduced well-being (in terms of a depressive state). The underlying causes might be complex (Büssing et al. 2017a, b), and thus to support persons experiencing these phases a thorough differentiation of the underlying ‘spirits’ is required. SDSs’ perceptions when these phases were overcome (i.e., greater spiritual serenity and depth or intentions to help others more) and also their strategies to cope with spiritual dryness will be presented later on.

## References

[CR1] Bech P, Olsen LR, Kjoller M, Rasmussen NC (2013). Measuring well-being rather than the absence of distress symptoms: A comparison of the SF-36 mental health subscale and the WHO-Five well-being scale. International Journal of Methods in Psychiatric Research.

[CR2] Büssing A, Büssing A, Dienberg T (2019). Geistliche Trockenheit bei Seelsorgern und Ordens-Christen. Geistliche Trockenheit - empirisch, theologisch, in der Begleitung.

[CR3] Büssing A, Baiocco F, Baumann K (2018). Spiritual dryness in Catholic lay persons working as volunteers is related to reduced life satisfaction rather than to indicators of spirituality. Pastoral Psychology.

[CR4] Büssing A, Baumann K, Jacobs C, Frick E (2017). Spiritual dryness in Catholic priests: internal resources as possible buffers. Psychology of Religion and Spirituality.

[CR5] Büssing A, Dienberg T (2019). Geistliche Trockenheit - empirisch, theologisch, in der Begleitung.

[CR6] Büssing A, Frick E, Jacobs C, Baumann Klaus (2016). Spiritual dryness in non-ordained Catholic pastoral workers. Religions.

[CR7] Büssing A, Günther A, Baumann K, Frick E, Jacobs C (2013). Spiritual dryness as a measure of a specific spiritual crisis in Catholic priests: Associations with symptoms of burnout and distress. Evidence-Based Complementary and Alternative Medicine.

[CR8] Büssing A, Rechia DR, Baumann K (2018). Validation of the gratitude/awe questionnaire and its association with disposition of gratefulness. Religions.

[CR9] Büssing A, Warode M, Gerundt M, Dienberg T (2017). Validation of a novel instrument to measure elements of Franciscan-inspired spirituality in a general population and in religious persons. Religions.

[CR10] Büssing A, Winter S, Baumann K (2020). Perception of religious brothers and sisters and lay persons that prayers go unanswered is a matter of perceived distance from God. Religions.

[CR11] Exline JJ, Grubbs JB, Homolka SJ (2015). Seeing God as cruel versus distant: Links with divine struggles involving anger, doubt, and fear of God’s disapproval. International Journal for the Psychology of Religion.

[CR12] Exline JJ, Rose ED, Paloutzian RF, Park CL (2013). Religious and spiritual struggles. Handbook of the psychology of religion and spirituality.

[CR13] Frick E, Büssing A, Baumann K, Weig W, Jacobs C (2016). Do self-efficacy expectation and spirituality provide a buffer against stress-associated impairmend of health? A comprehensive analysis of the German Pastoral Ministry Study. Journal of Religion and Health.

[CR14] Ignatius of Loyola. (1914). *The spiritual exercises of St. Ignatius of Loyola*. (Father E. Mullan, Trans.). Christian Classics Ethereal Library. http://www.ccel.org/ccel/ignatius/exercises.html.

[CR15] Jamison, C. (2008). Spiritual apathy: The forgotten deadly sin. Thinking faith 2008. Online available https://www.thinkingfaith.org/articles/20081009_1.htm.

[CR16] John of the Cross. (1959). *Dark night of the soul* (E. Allison Peers, Trans.). Christian Classics Ethereal Library. 1994. https://ccel.org/ccel/j/john_cross/dark_night.html.

[CR17] John of the Cross. (1962). *Ascent of mount Carmel* (E. Allison Peers, Trans.). Christian Classics Ethereal Library. 1994. https://ccel.org/ccel/j/john_cross/ascent.html.

[CR18] Johnsson WG (2017). Where are we headed?—adventism after San Antonio.

[CR31] Knight GR (1989). Angry saints: Tensions and possibilities in the adventist struggle over righteousness by faith.

[CR19] Maselko J, Kubzansky LD (2006). Gender differences in religious practices, spiritual experiences, and health: Results from the US General Social Survey. Social Science and Medicine.

[CR20] Morton KR, Lee JW, Martin LR (2017). Pathways from religion to health: Mediation by psychosocial and lifestyle mechanisms. Psychology of Religion and Spirituality.

[CR21] Orlich MJ, Singh PN, Sabaté J, Jaceldo-Siegl K, Fan J, Knutsen S, Beeson WL, Fraser GE (2013). Vegetarian dietary patterns and mortality in Adventist Health Study 2. JAMA Internal Medicine.

[CR22] Pontikos E (2007). Über die acht Gedanken.

[CR23] Rahner K (1993). Meine Nacht kennt keine Finsternis [My night knows no darkness].

[CR24] Stauner N, Exline JJ, Pargament KP (2016). Religious and spiritual struggles as concerns for health and well-being. Horizonte, Belo Horizonte.

[CR150] Taylor RJ, Chatters LM, Levin JS (2001). Religion in the lives of African Americans: Social, psychological, and
health perspectives.

[CR26] Underwood LG (2011). The daily spiritual experience scale: Overview and results. Religions.

[CR30] Underwood LG, Teresi JA (2002). The daily spiritual experience scale: Development, theoretical description, reliability, exploratory factor analysis, and preliminary construct validity using health-related data. Annals of Behavioral Medicine.

[CR27] Vine, C. (2014). Applying the biblical practice of meditation among adventist frontier mission employees. Project Document Andrews University Seventh-day Adventist Theological Seminary, Corpus ID: 74309650. https://www.semanticscholar.org/paper/Applying-the-Biblical-Practice-of-Meditation-Among-Vine/ed96fc4261c4d8b8c7913b388c04490e581f4e86#paper-header.

[CR28] White EG (1868). Testimonies for the Church I (1T 576.2).

[CR29] White EG (1892). Steps to Christ.

